# Association of tumor necrosis factor-α (TNF-α) gene promoter polymorphisms with aggressive and chronic periodontitis in the eastern Indian population

**DOI:** 10.1042/BSR20171212

**Published:** 2018-07-31

**Authors:** Poulami Majumder, Keheibamding Thou, Mandar Bhattacharya, Vineet Nair, Sujay Ghosh, Subrata Kumar Dey

**Affiliations:** 1 Department of Biotechnology, Centre for Genetic studies, School of Biotechnology and Biological Sciences, West Bengal University of Technology, BF-142, Sector I, Salt Lake City, Kolkata, West Bengal 70064, India; 2 Department of Periodontia, North Bengal Dental College, Siliguri, West Bengal, 734012, India; 3 Department of Zoology, Cytogenetics and Genomics Research Unit, University of Calcutta, 35 Ballygunge Circular Road, Kolkata, West Bengal 700019, India

**Keywords:** allele, genotype, Periodontitis, PCR-sequencing, SNP, TNF-α

## Abstract

**Background:** Periodontitis is a very common inflammatory oral disease. Tumor necrosis factor-α (TNF-α) is a cytokine that has been involved with the gingival tissue destruction and remodeling occurrence. We investigated the association of single nucleotide polymorphisms (SNPs) in TNF-α gene promoter region with the susceptibility of aggressive and chronic periodontitis in the eastern Indian population.

**Methods:** A total of 397 DNA samples from venous blood were isolated. 40 individuals were aggressive periodontitis patients, 157 were identified chronic periodontitis patients, and the remaining 200 were healthy individuals. Five SNPs of TNF-α at promoter region (rs361525, rs1800629, rs1799724, rs1800630, and rs1799964) were genotyped by PCR-sequencing in periodontitis patients and control subjects.

**Results:** rs1800629 (-308G/A) polymorphism was more frequent in both aggressive and chronic periodontitis patients compared with the control population, though the allele frequency was different only in aggressive periodontitis patients. On the other hand, both the genotypic and allelic variation of rs361525 (-238G/A) polymorphism were found significantly less frequently in aggressive and chronic periodontitis than in controls. The other polymorphisms like rs1799724 (-857C/T) and rs1799964 (-1031T/C) were significantly different between chronic periodontitis patients and control subjects.

**Conclusion:** The findings suggest that the rs1800629 (-308G/A) polymorphism of TNF-α gene is associated with both aggressive and chronic periodontitis while rs1799724 (-857C/T) and rs1799964 (-1031T/C) polymorphisms of TNF-α gene is associated only with the increased susceptibility to chronic periodontitis.

## Introduction

Innumerable diseases associated with tooth loss and oral inflammation account for an overwhelming phenomenom in the developing world. There are various known factors associated with tooth loss but periodontitis seems to be considered a main offender. Periodontitis is the most frequent disease of tooth and associated tissues. More than 90% of the worldwide population is affected by chronic periodontitis (CP), a common type of periodontitis [1]. CP is a chronic inflammatory infectious disease leading to the destruction of tooth-supporting tissues, caused by degradation of the extracellular matrix [2]. A number of studies have been carried out to solve the complex etiology of CP, regarding genetics, molecular biology, microbiology, and other relevant areas. Some pathogens are the external initiating factor in the pathogenesis of CP, but CP might be influenced by other factors. These factors may not cause disease directly, but can modify its severity [3]. Among these factors, genetic alterations called polymorphisms, are commonly found in populations [4]. Another severe type of periodontitis, aggressive periodontitis (AgP), results in rapid rate of bone destruction and attachment loss. Previously, it was known as “early-onset periodontitis” or “juvenile periodontitis” [5]. AgP patients do not show large accumulation of plaque and/or calculus, an in these cases genetics are often responsible. Adolescents are affected more with AgP than other age groups [6]. AgP is considered to be a more complex genetic disease than CP as its degrees of harmful risk are unpredictable. Host heterogeneity may be considered as conclusive factor in the pathogenesis of AgP and the influence of genes and environmental factors determine the patient’s phenotypes accordingly [7]. The overall causes of periodontitis are very complex due to various exogenous factors and yet to be identified genetic factors [5], but the major factors contributing to periodontitis are genetic, environmental/behavioral and host-related.

A complex network of cytokines is thought to be involved in this inflammatory disease and plays a crucial role in the regulation of periodontal tissue health [8]. Tumor necrosis factor-α (TNF-α) is one of the most potent proinflammatory cytokines that plays a major role in tissue injury and induces the bone resorption in the immune system [9]. TNF-α triggers various immune and inflammatory process, activation of cells, adhesion of polymorphonuclear leukocytes (PMNs) to endothelial cells [10], phagocyte activation and ICAM-1 expression [11], as well as having roles in necrosis and apoptosis. Bacterial pathogens of dental plaque stimulate the secretion of TNF-α which causes osteoclast differentiation and results in bone resorption [12]. TNF-α promotes the release of matrix metalloproteinase enzymes (MMPs) that destroy the extracellular matrix of gingiva [13] and initiate the rapid disease progression. Genetic analysis has revealed a large number of single nucleotide polymorphic (SNP) sites within the TNF-α locus that are related to periodontitis [14]. The TNF-α gene is located at short (p) arm of chromosomes 6 at position 21.3 encoding a multifunctional proinflammatory cytokine that is secreted mainly by macrophages. Cytokine-producing genes exhibit polymorphisms, which modify synthesis of proinflammatory cytokines such as TNF-α [15].

Scientists stipulated that approximately 50% of the clinical differences of periodontal disease evolved from gene polymorphisms [16]. Therefore, many gene polymorphisms including TNF-α have been investigated [4,6,17–22]. The human genome is arranged in a complete set of nucleotide bases, any single base change can be easily identified, helping to determine genetic association with susceptibility to periodontal disease. Various studies related to SNPs of the TNF-α gene show different level of association with this disease in their different populations [4,6,14,23–25]. These polymorphisms may also cause a change in protein expression resulting in possible clinical outcomes of this disease which determines the susceptibility to periodontitis [26]. TNF-α gene polymorphisms at the promoter region have been intensively studied and some of them were shown to be associated with the severity of periodontitis. In addition, there is a considerable numbers of studies that have been made to investigate the association of TNF-α gene polymorphisms in different ethnicities as well as in different populations [8,21,22]. Therefore, the TNF-α gene polymorphisms play a pivotal role in periodontitis susceptibility. We hypothesized that the genetic variation affecting the expression or activity of TNF-α influences the susceptibility and severity of periodontal disease.

The aims of our study are

1. to find out the association of TNF-α gene polymorphisms with both chronic and aggressive periodontal disease in the Indian population;
2. to analyze the linkage disequilibrium and haplotype distribution in both diseased populations – CP and AgP;
3. to explore the effect of epidemiological factors on periodontitis, if these factors are operative in our studied population.

In the Indian population, especially the eastern Indian people with periodontitis, to the best of our knowledge, the present study is the first to evaluate polymorphisms of TNF-α. Previously, we have analyzed MMP-9 gene polymorphisms in association with periodontitis in the same population [27]. The present study sought to investigate the effect of TNF-α (-238G/A, -308 G/A, -857C/T, -863C/A, and -1031 T/C) gene promoter SNPs on susceptibility to periodontitis in the Indian population.

## Materials and methods

### Study subjects

The study was carried out with 397 individuals: 40 individuals were aggressive periodontitis (AgP) patients, 157 patients with chronic periodontitis (CP), and 200 individuals were identified as periodontal healthy controls (HC). Most of the participants resided in the eastern region of India, though some of participants were from northeastern region. Those experimental subjects were recruited between September 2014 and January 2016. The study was approved by the institutional ethics committee. Participants eligible for the study gave their written consent in accordance with Helsinki Declaration & ICMR (Indian Council of Medical Research) guidelines and after being informed about the purpose of the study. In addition, patients from whom consent could not be obtained were excluded from the study. Clinical and epidemiological data were taken; those who had systemic diseases which could modify the periodontal status (viz. the cerebrovascular disease, arthrosclerosis, hypertension, coronary heart disease etc.) were excluded from the present study. The inclusion criteria of our study for the both AgP and CP patients were that they must have at least ten remaining teeth and showing probing depth (PD) and clinical attachment loss (CAL) more than 3 mm. All study subjects were in age range 15–65 years. We determined the smoking status by interviewing them and made smoker and nonsmoker groups in both patients and the control group. Those who smoked ≥10 cigarettes per day for 5 years were identified as smokers in case of all groups (viz. AgP, CP, and HC) whereas nonsmokers were who had not smoked for the last 5 years [28]. Chewing-tobacco users were also identified by defining their tobacco usage ≥3 times per day for 5 years. The status of tea intake is divided into three categorie: more than four cups per day; less than four cups per day and no intake. Table 1 shows the sociodemographic data of the patient and healthy control groups. All CP and AgP patients were diagnosed according to their physical, medical and dental history, tooth mobility, and radiographs. Clinical parameters included PD, CAL, plaque index (PI), and gingival index (GI) (Table 2). Clinical assessment of study subjects was as follows: AgP and CP subjects with signs of clinical inflammation consistent with local etiological factors, GI score > 1, PD ≥ 4 mm, CAL ≥ 4 mm, with radiographic evidence of bone loss were included in the study [29]. AgP subjects with noncontributory medical history, rapid attachment loss and bone destruction, familial aggregation of cases and amount of deposits which are inconsistent with the severity of periodontal tissue destruction. HC subjects have a “healthy periodontium” with no evidence of loss of connective tissue attachment or supporting bone or other signs of disease activity.

**Table 1:** Sociodemographic analysis in AgP, CP, and HC

Parameters	AgP (*n*=40) (%)	CP (*n*=157) (%)	HC (*n*=200) (%)	AgP vs HC (*P* value)	CP vs HC (*P* value)
Age					
Age range (years)	17–44	22–69	24–65		
Mean (±SD)	30.23 ± 6.81	41.59 ± 11.12	38.41 ± 9.48	0.0001*	0.0038*
Gender (%)					
Male	60.0	65.33	47.5	0.1515	0.0016*
Female	40.0	34.67	52.5	Ref	Ref
Ethnicity (%)					
Hindu	60.0	44.67	46.0	0.1085	0.6123
Muslims	40.0	55.33	54.0	Ref	Ref
Smoker (%)					
Yes	25	62.0	20.5	0.5261	<0.0001*
No	75	38.0	79.5	Ref	Ref
Tobacco Chewer (%)					
Yes	7.5	62.67	49.5	0.0001*	<0.0001*
No	92.5	37.33	50.5	Ref	Ref
Tea Drinker (%)					
≥4 cups/day	15.0	44.67	31.0	0.2479	<0.0001*
≤4 cups/day	57.5	46.0	38.5	0.2124	0.0001*
Never	27.5	9.33	30.5	Ref	Ref

* Statistically significant (*P*<0.05).

**Table 2:** Clinical parameters in three study groups

Parameters	AgP	CP	HC
PD (mm) (full mouth mean ± SD)	6.01 ± 1.94*	6.36 ± 1.62*	0.34 ± 0.66
CAL (mm) (full mouth mean ± SD)	8.3 ± 2.12*	8.79 ± 1.94*	0.03 ± 0.21
PI	2.83 ± 1.08*	2.95 ± 0.81*	0.05 ± 0.2
GI	3.05 ± 0.85*	2.61 ± 1.01*	0.01 ± 0.08

* Statistically significant (*P*<0.05).

### Genotyping

Genomic DNA from each individual was isolated from 4 ml of ethylenediaminetetraacetic acid (EDTA)-anticoagulated peripheral blood samples by Genomic DNA Mini Kit (DSRGT DNA Isolation Kit, India) based on the instructions of the protocol. Extracted DNA was labeled and stored in TE buffer at −20°C until use. To determine the five different TNF-α promoter gene polymorphisms (-238G/A, -308 G/A, -857C/T, -863C/A, and -1031 T/C), polymerase chain reaction (PCR) was performed. All PCRs were carried out in 50 µl containing 0.1 µg of DNA, 5 µl of 10× buffer (Invitrogen®, Sao Paulo Brazil), 5 µl of 0.5 mM MgCl_2_ (Invitrogen®), 1 µl of 10 mM dNTPs (Himedia®, India), 1 μl of 0.5 µM of each primer (Sigma-Aldrich®, India), and 2.5 U of Taq DNA polymerase (Invitrogen®). The cycling parameters for the amplification of those five polymorphisms of TNF-α gene are detailed in Table 3. PCR was performed with a Thermal cycler (Applied Biosystems). The -238G/A (rs361525) polymorphism of the TNF-α gene was amplified by PCR with a set of primers, F-5′CAGTGGGGTCTGTGAATTCC3′; R-5′TCCCTCTTAGCTGGTCCTCT3′. The -308 G/A (rs1800629) polymorphism was determined by F-5′CAGTGGGGTCTGTGAATTCC3′; R-5′GGGCGGGGAAAGAATCATTC3′ primers. The -857C/T (rs1799724) polymorphism was analyzed by F-5′CTGCTTGTGTGTGTGTGTCT3′; R-5′ CCGGAGACTCATAATGCTGGT3′ primers. The -863C/A (rs1800630) and -1031T/C (rs1799964) were analyzed by F-5′GTGTGTGTCTGGGAGTGAGA3′; R-5′ GCAGGCCTTCTTCTTTCATTCT3′ primers and F-5′GAGAGAAAGAAGTAGGCATGAGG3′; R-5′TCTTAAACGTCCCCTGTATTCCA3′ primers respectively (Table 3). The amplified products were electrophoresed on a 2–3% agarose gel. All PCR products were sequenced (Sanger method of sequencing) by Prism 3100 DNA Genetic Analyzer (Applied Biosystems, Carlsbad, CA, U.S.A.).

**Table 3:** Primers and PCR condition

TNF-α gene polymorphisms	Primer	PCR condition	Product size
**rs361525 -238G** >**A**	F-5′CAGTGGGGTCTGTGAATTCC3′	1 cycle 96°C for 6 min; 30 cycles (96°C for 30 s, 58°C for 1 min, 72°C for 1 min); final extension at 72°C for 7 min	688 bp
	R-5′ TCCCTCTTAGCTGGTCCTCT3′		
**rs1800629 -308G**>**A**	F-5′CAGTGGGGTCTGTGAATTCC3′	1 cycle 96°C for 6 min; 30 cycles (96°C for 30 s, 56°C for 1 min, 72°C for 1 min); final extension at 72°C for 5 min	593 bp
	R-5′ GGGCGGGGAAAGAATCATTC3′		
**rs1799724 -857C**>**T**	F-5′CTGCTTGTGTGTGTGTGTCT3′	1 cycle 96°C for 6 min; 30 cycles (96°C for 30 s, 59°C for 1 min, 72°C for 1 min); final extension at 72°C for 7 min	572 bp
	R-5′ CCGGAGACTCATAATGCTGGT3′		
**rs1800630 -863C**>**A**	F-5′GTGTGTGTCTGGGAGTGAGA3′	1 cycle 96°C for 6 min; 30 cycles (96°C for 30 s, 57°C for 1 min, 72°C for 30 s); final extension at 72°C for 7 min	581 bp
	R-5′ GCAGGCCTTCTTCTTTCATTCT3′		
**rs1799964 -1031T**>**C**	F-5′GAGAGAAAGAAGTAGGCATGAGG3′	1 cycle 96°C for 6 min; 30 cycles (96°C for 30 s, 59°C for 30 s, 72°C for 1 min); final extension at 72°C for 7 min	600 bp
	R-5′TCTTAAACGTCCCCTGTATTCCA3′		

### Statistical analysis

The distribution of five TNF-α genotypic frequencies, variant allele carriage, and allelic frequencies for each group was calculated by using a chi square (χ^2^) test. Allelic frequencies were analyzed from the observed number of genotypes. 2 × 2 contingency tables were constructed for a chi square test (Pearson chi square test, likelihood ratio) that was performed to justify the statistical significance of genotypic differences between patients and the control group. To justify the distribution of genotypic frequencies, a Hardy–Weinberg equilibrium test was done with χ^2^ critical value. The odds ratio (OR) was estimated with a 95% confidence interval (95% CI) and a probability value (*P* value) of less than 5% was considered to be statistically significant i.e. *P*<0.05. The difference between clinical parameters was assessed by one-way ANOVA test. Age, gender, ethnicity, smoking status, chewing-tobacco status, and tea-drinking habits were used as independent variables for multiple logistic regression analysis. All statistical analyses were performed by commercially available software (Statistical Package for Social Sciences, version 16.0 for windows, SPSS Inc., Chicago, IL, U.S.A.). Moreover, we also studied five different SNPs in the promoter region of the TNF-α gene on chromosome no. 6. To assess the multi loci-based genetic association of a complex disease like periodontitis we used SHEsis, an online tool for analyses of linkage disequilibrium (LD) between markers and haplotype distributions (find more at http://analysis.bio-x.cn). A *P* value of <0.05 was determined to be statistically significant.

## Results

### Sociodemographic and clinical characteristics of study subjects

The sociodemographic characteristics and clinical backgrounds are presented in Tables 1 and 2 respectively. The mean age of the AgP, CP and HC groups were 30.23 ± 6.81 years, 41.59 ± 11.12 years, and 38.41 ± 9.48 years respectively (Table 1). In the CP group, significant differences were found in the distribution of categories such as gender, smoker, chewing-tobacco user, and drinking tea. It was also found that the AgP group showed a significant difference only in thge chewing tobacco category (*P*<0.05). Clinical parameters like PD, CAL, PI, and GI were significantly higher in both diseased groups by one-way ANOVA analysis (*P*<0.0001) (Table 2).

### SNP distribution of TNF-α promoter region in the study subjects

**Figure 1:**
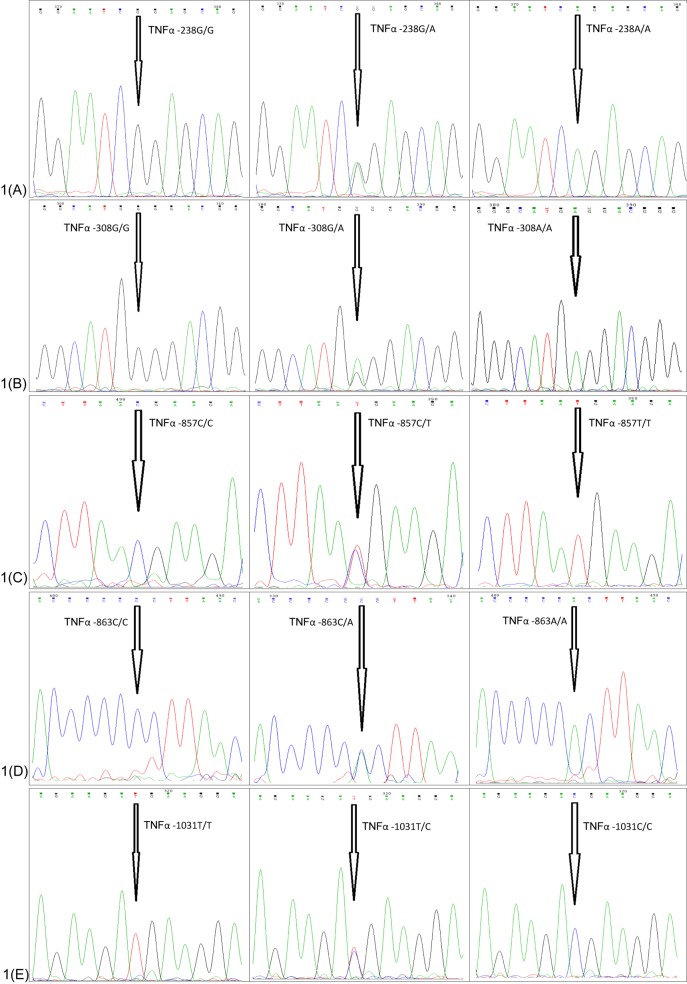
The chromatograms show nucleotide change at five different SNPs of TNF-α (**A**) shows -238G/A variants; (**B**) shows -308G/A variants; (**C**) shows -857C/T variants; (**D**) shows -863C/A variants; and (**E**) shows -1031T/C variants.

The frequencies of all TNF-α (-238G/A, -308 G/A, -857C/T, -863C/A, and -1031 T/C) genotypes in the AgP groups and the -857C/T, -863C/A, and -1031 T/C genotypes in the CP group were found to be in agreement with the Hardy–Weinberg equilibrium (*P*>0.001, χ^2^<10.83). However, the distribution of the -238G/A and -308 G/A genotypes in both the CP and HC groups differed from the Hardy–Weinberg law (*P*<0.001, χ^2^>10.83). The genotype distribution, allele frequencies of TNF-α promoter gene polymorphisms in the AgP, CP, and HC groups are presented in Table 4. Chromatograms of the five different TNF- α polymorphisms are shown in Figure 1A–E.

**Table 4:** TNF-α genotype and allelic frequencies in AgP, CP, and HC group

Alleles and genotyping	AgP *N*=40 (%)	CP *N*=157 (%)	HC *N*=200 (%)	Odds ratio		95% CI		*P* value		Chi square (Pearson *P* value)	
				AgP vs HC	CP vs HC	AgP vs HC	CP vs HC	AgP vs HC	CP vs HC	AgP vs HC	CP vs HC
**rs361525 -238G >A**											
GG	26	86	125								
GA	12	47	44	1.31	1.55	0.609–2.819	0.947–2.545	0.306	0.053	0.48 (0.488)	3.06 (0.081)
AA	2	24	31	0.31	1.13	0.069–1.377	0.617–2.049	0.081	0.406	2.61 (0.106)	0.15 (0.698)
A allele carriage	14	71	75	0.89	1.37	0.441–1.825	0.899–2.104	0.456	0.086	0.09 (0.764)	2.17 (0.14)
G	64	219	294								
A	16	95	95	0.69	1.2	0.384–1.252	0.867–1.669	0.139	0.153	0.49 (0.222)	1.23 (0.267)
**rs1800629 -308G>A**											
GG	10	40	101								
GA	15	56	55	2.75	2.57	1.16–6.54	1.525–4.333	<0.01*	<0.001*	5.56 (0.018)	12.84 (<0.001)
AA	15	61	44	3.44	3.51	1.43–8.26	2.054–5.966	<0.001*	<0.001*	8.28 (<0.001)	21.98 (<0.001)
A allele carriage	30	117	99	3.06	2.98	1.42–6.59	1.896–4.696	0.002 *	<0.001*	8.72 (0.003)	23.05 (<0.0001)
G	35	136	257					.			
A	45	178	143	2.31	2.35	1.42–3.761	1.737–3.183	<0.0001*	<0.0001*	11.76 (<0.0001)	31.16 (<0.0001)
**rs1799724 -857C>T**											
CC	21	55	97								
CT	16	84	81	0.912	1.83	0.446–1.863	1.166–2.869	0.473	0.009*	0.06 (0.806)	6.97 (0.008)
TT	3	18	22	0.629	1.44	0.173–2.301	0.712–2.921	0.355	0.2001	0.49 (0.481)	1.04 (0.307)
T allele carriage	19	102	103	0.852	1.75	0.431–1.68	1.13–2.68	0.386	0.007 *	0.21 (0.646)	6.53 (0.0106)
C	58	194	275								
T	22	120	125	0.83	1.36	0.489–1.423	0.997–1.856	0.301	0.03*	0.44 (0.507)	3.79 (0.052)
**rs1800630 -863C>A**											
CC	17	84	104								
CA	19	58	66	1.76	1.08	0.854–3.63	0.69–1.715	0.087	0.402	2.39 (0.122)	0.13 (0.718)
AA	4	15	30	0.85	0.62	0.255–2.608	0.312–1.225	0.491	0.111	0.12 (0.731)	1.91 (0.166)
A allele carriage	23	73	96	1.46	0.94	0.738–2.909	0.619–1.431	0.177	0.43	1.2 (0.273)	0.08 (0.777)
C	53	226	274								
A	27	88	126	1.12	0.85	0.666–1.843	0.612–1.171	0.392	0.177	0.16 (0.689)	1.01 (0.315)
**rs1799964 -1031T>C**											
TT	13	38	88								
TC	21	57	47	3.02	2.81	1.391–6.578	1.633–4.83	0.004*	<0.0001*	8.2 (0.004)	14.28 (<0.0001)
CC	6	62	65	0.63	2.2	0.225–1.731	1.319–3.699	0.256	0.001*	0.83 (0.362)	9.21 (0.002)
C allele carriage	27	119	112	1.63	2.46	0.795–3.346	1.553–3.896	0.12	<0.0001*	1.81 (0.178)	15.09 (<0.0001)
C	47	133	223								
T	33	181	177	0.88	1.71	0.543–1.439	1.272–2.311	0.357	<0.0001*	0.24 (0.624)	12.62 (0.0003)

* Statistically significant (*P*<0.05).

#### -238G/A genotypic and allelic distribution

There was no significant difference between the disease (AgP and CP) and control groups in the TNF-α -238G/A genotypes and allelic distribution. When the G/A and A/A genotypes were combined, the frequency of the A/A carriers in both diseased groups was significantly lower compared with the control group.

#### -308G/A genotypic and allelic distribution

There were significant differences in the distribution of genotypes (*P*<0.001) and allele frequencies (*P*<0.001) of -308G/A polymorphism between both the AgP vs HC and the CP vs HC groups. The AgP and CP patients had a higher frequencies (37.5% and 38.8% respectively) of the A/A genotype than the HC group (22%). The frequency of the A allele was higher in the diseased groups (56.3% in AgP and 56.7% in CP) compared with 35.7% in the HC group. When the A/A and G/A genotypes were combined, the frequency of the A carriers was significantly higher compared with the healthy group (75% and 74.5% vs 49.5%; *P*=0.002, OR = 3.06, 95% CI = 1.42–6.59 and *P*<0.0001, OR = 2.98, 95% CI = 1.896–4.696).

#### -857C/T genotypic and allelic distribution

There was a significant difference in the distribution of the C/T genotypes (*P*=0.009, OR = 1.83, 95% CI = 1.16–2.86) of the -857C/T polymorphism in the CP group in compared with the HC groups, while the T/T genotypes in the same group had no significant difference (*P*=0.2, OR = 1.44, 95% CI = 0.712–2.921). The frequency of the T allele was higher in the CP groups (38.3%) compared with 31.2% in the HC group (*P*=0.03, OR = 1.36, 95% CI = 0.997–1.856). When the T/T and C/T genotypes were combined, the frequency of the T carriers was significantly higher in the CP group than in the healthy group (64.9% vs 51.5%, *P*=0.007, OR = 1.75, 95% CI = 1.13–2.68). On the other hand, there was no significant difference in genotypic, allelic, and rare allele carriage frequency in the AgP group in compared with the HC group.

#### -863C/A genotypic and allelic distribution

There was no significant difference between disease groups (AgP and CP) and control groups in the genotypes and allelic distribution of the TNF-α -863C/A polymorphism. When the C/A and A/A genotypes were combined, the frequency of the A/A carriers in both diseased groups was significantly lower compared with the control group.

#### -1031T/C genotypic and allelic distribution

There were significant differences in the distribution of T/C genotypes of -1031T/C polymorphism in both diseased groups (in AgP: *P*=0.004, OR = 3.02, 95% CI = 1.391–6.578; in CP: *P*<0.0001, OR = 2.81, 95% CI = 1.633–4.83), while the frequency of the C/C genotypes was lower in the AgP group and higher in the CP group (15% in AgP and 39.5% in CP) compared with 32.5% in the HC group (in AgP: *P*=0.256, OR = 0.63, 95% CI = 0.225–1.731; in CP: *P*=0.001, OR = 2.2, 95% CI = 1.319–3.699). The allelic frequency of C allele in CP group was significantly higher (57.6%) than the HC groups (44.3%) (*P*=0.0001, OR = 1.71, 95% CI = 1.272–2.311), but in the AgP group it was lower (41.3%; *P*=0.357, OR = 0.88, 95% CI = 0.543–1.439) compared with the control. When the T/C and C/C genotypes were combined, the frequency of the C carriers was significantly higher in the CP groups compared with the healthy group (75.8% vs 56%, *P*<0.0001, OR = 2.46, 95% CI = 1.553–3.896).

### Haplotype frequency and linkage disequilibrium analysis

**Figure 2:**
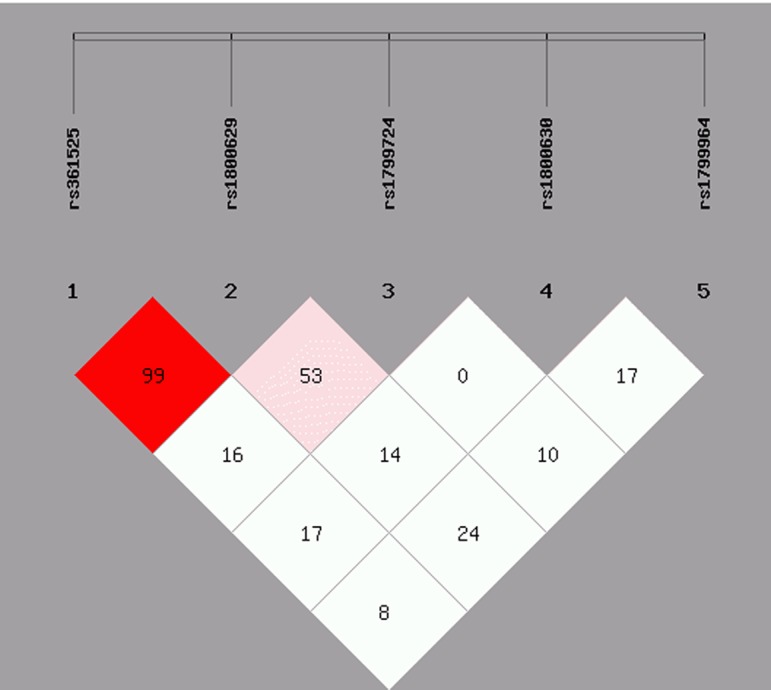
Linkage disequilibrium (LD) pattern of TNF-α genetic variants in the AgP population The results represent the strong LD (D0 > 0.8) of TNF-α -238G/A, rs361525 and -308G/A, rs1800629.

**Figure 3:**
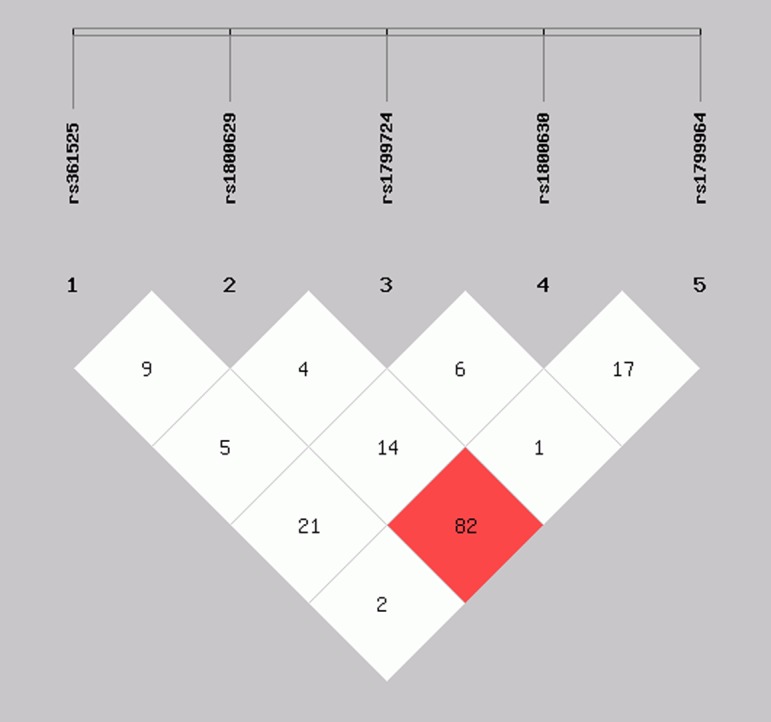
Linkage disequilibrium (LD) pattern of TNF-α genetic variants in the CP population The results represent the strong LD (D0 > 0.8) of TNF-α -308G/A, rs1800629 and -1031T/C, rs1799964.

Based on the findings revealed from the five SNPs in TNF-α and the determined linkage disequilibrium (LD), our study highlighted that two SNPs, i.e. -238G/A and -308G/A, were linked with one another (D > 0.8) (Figure 2) in the AgP population, and -308G/A and -1031T/C SNPs were linked in the CP population (D > 0.8) (Figure 3). As shown in Tables 5 and 6, our study highlighted that a two haplotypes (GGCCC and AATCC) increased the susceptibility of both AgP and CP compared with the control population (In AgP: OR = 14.652, 95% CI = 3.48–61.612, *P*=0.002; OR = 4.54, 95% CI = 0.927–22.246, *P*=0.041 respectively and In CP: OR = 7.885, 95% CI = 2.08–29.54, *P*=0.0003; OR = 5.448, 95% CI = 1.599–18.569, *P*=0.002 respectively). The other haplotypes (GACAC, AATAC, GATAC, GGCCT, GGTAT, and GGTCT) were not to be found significantly associated with the increased susceptibility of both diseased groups. There were some other haplotypes like GACCT, GGCAC, and GGCAT that were significantly associated with the increased susceptibility in AgP groups whereas GATCC, AGTCT, AACCC, and AACAC haplotypes were associated only with increased risk of CP.

**Table 5:** Haplotype distribution in AgP group (as derived from SHEsis program)

Haplotypes	Frequency		OR (95% CI)	*P* value
	AgP (%)	HC (%)		
GACAC	4.13 (0.052)	12.85 (0.032)	1.57 (0.503–4.896)	0.433
AATCC*	2.99 (0.037)	3.26 (0.008)	4.54 (0.927–22.246)	0.041
AATAC	2.22 (0.028)	21.52 (0.054)	0.48 (0.118–1.952)	0.294
GACCC	7.64 (0.096)	40.38 (0.101)	0.89 (0.396–2.026)	0.792
GACCT*	6.32 (0.079)	3.99 (0.01)	8.18 (2.27–29.39)	0.0001
GATAC	3.16 (0.04)	47.42 (0.119)	0.29 (0.09–0.932)	0.027
GGCAC*	3.93 (0.049)	2.15 (0.005)	9.16 (1.71–49.19)	0.0018
GGCAT*	7.54 (0.094)	8.16 (0.02)	4.804 (1.73–13.36)	0.001
GGCCC*	7.29 (0.091)	2.61 (0.007)	14.652 (3.48–61.612)	0.0021
GGCCT	12.09 (0.15)	148.03 (0.37)	0.277 (0.145–0.53)	0.0047
GGTAT	2.69 (0.034)	8.83 (0.022)	1.47 (0.368–5.91)	0.581
GGTCT	1.43 (0.018)	13.20 (0.033)	0.511 (0.089–2.92)	0.442

* Statistically significant (*P*<0.05).

**Table 6:** Haplotype distribution in CP group (as derived from SHEsis program)

Haplotypes	Frequency		OR (95% CI)	*P* value
	CP (%)	HC (%)		
GACCC	37.15 (0.118)	40.38 (0.101)	1.26 (0.785–2.02)	0.335
GATAC	15.65 (0.05)	47.42 (0.119)	0.408 (0.226–0.739)	0.002
GATCC*	37.3 (0.119)	4.54 (0.011)	12.46 (4.644–33.438)	0.00004
GGCAT	11.53 (0.037)	8.16 (0.02)	1.925 (0.775–4.782)	0.152
GGCCC*	14.69 (0.047)	2.61 (0.007)	7.885 (2.08–29.54)	0.0003
GGCCT	41.13 (0.131)	148.03 (0.37)	0.265 (0.18–0.39)	0.000005
GGTAT	10.58 (0.034)	8.83 (0.022)	1.623 (0.655–4.02)	0.291
GGTCT	14.38 (0.046)	13.20 (0.033)	1.479 (0.689–3.172)	0.313
GACAC	16.06 (0.051)	12.85 (0.032)	1.709 (0.807–3.619)	0.157
AGTCT*	11.07 (0.035)	5.86 (0.015)	2.582 (0.937–7.112)	0.057
AGTAT	7.90 (0.025)	12.09 (0.03)	0.869 (0.35–2.159)	0.762
AGCCT	16.4 (0.052)	42.68 (0.107)	0.484 (0.268–0.874)	0.014
AATCC*	12.81 (0.041)	3.26 (0.008)	5.448 (1.599–18.569)	0.002
AATAC	1.94 (0.006)	21.52 (0.054)	0.115 (0.026–0.501)	0.0005
AACCC*	22.73 (0.072)	7.13 (0.018)	4.54 (1.929–10.687)	0.0001
AACAC*	14.34 (0.046)	2.85 (0.007)	7.012 (1.95–25.218)	0.0005

* Statistically significant (*P*<0.05).

### Logistic regression analysis

Since the pathogenesis of periodontitis is multifactorial, multiple logistic regression analysis was used to evaluate the associations of the rare allele carrying genotypes with AgP and CP susceptibility, while adjusting for the epidemiological variables such as a subject age, gender and smoking status to control for possible confounding effects in the present study (Table 7). The subjects’ gender and smoking and chewing-tobacco status were found to be significantly associated (*P*<0.05) with CP (AOR = 2.363, 95% CI = 1.185–4.715, *P*=0.01; AOR = 10.057, 95% CI = 4.962–20.38, *P*<0.0001 and AOR = 3.701, 95% CI = 2.21–6.196, *P*<0.0001 respectively). The TNF-α -308 A allele carrying genotype was found to be significantly associated with both the AgP and CP groups (AOR = 3.607, 95% CI = 1.49–8.732, *P*=0.004 and AOR = 2.805, 95% CI = 1.641–4.794, *P*<0.0001 respectively). While the other SNP, i.e. -1031 C, allele carrying genotypes were found to be significant only for CP after adjustment for age, gender, smoking and chewing tobacco status, and habit of drinking tea (AOR = 2.537, 95% CI = 1.483–4.342, *P*=0.001). The TNF-α -238 A, -857 T and -863 A allele carrying genotypes were not found to be significantly associated with AgP (AOR = 1.654, 95% CI = 0.668–4.1, *P*=0.277; AOR = 0.782, 95% CI = 0.356–1.72, *P*=0.541 and AOR = 1.481, 95% CI = 0.679–3.23, *P*=0.324 respectively) as well as CP (AOR = 1.348, 95% CI = 0.81–2.245, *P*=0.251; AOR = 1.423, 95% CI = 0.858–2.36, *P*=0.172 and AOR = 0.787, 95% CI = 0.476–1.301, *P*=0.35 respectively).

**Table 7:** Logistic regression analysis of epidemiological factors in AgP and CP groups distributed by rare allele carrying and noncarrying genotypes

Parameters	AgP vs HC			CP vs HC		
	AOR	95% CI	*P* value	AOR	95% CI	*P* value
Age	0.865	0.812–0.922	<0.0001	1.019	0.995–1.04	0.139
Gender						
Male	0.57	0.235–1.386	0.215	2.363	1.185–4.715	0.015*
Female						
Ethnicity						
Hindu	2.007	0.904–4.455	0.087	0.699	0.421–0.161	0.167
Muslim						
Smoker	1.346	0.473–3.832	0.578	10.057	4.962–20.38	<0.0001*
Tobacco Chewer	0.225	0.063–0.804	0.022	3.701	2.21–6.196	<0.0001*
Tea Drinker						
≥4 cups/day	1.913	0.663–5.522	0.23	1.236	0.709–2.152	0.455
≤4 cups/day	1.042	0.307–3.539	0.948	0.39	0.187–0.812	0.012
Never						
-238 A allele carriage						
Noncarrier						
Carrier	1.654	0.668–4.1	0.277	1.348	0.81–2.245	0.251
Age	0.867	0.812–0.926	<0.0001	1.02	0.995–1.045	0.123
Gender						
Male	0.556	0.216–1.432	0.224	2.354	1.18–4.695	0.015*
Female						
Ethnicity						
Hindu	1.794	0.76–4.237	0.183	0.689	0.414–1.146	0.151
Muslim						
Smoker	1.061	0.362–3.106	0.914	9.879	4.871–20.037	<0.0001*
Tobacco Chewer	0.341	0.092–1.265	0.108	3.631	2.165–6.089	<0.0001*
Tea Drinker						
≥4 cups/day	1.96	0.655–5.867	0.229	1.234	0.708–2.151	0.458
≤4 cups/day	1.19	0.332–4.335	0.782	0.378	0.181–0.789	0.01
Never						
-308 A allele carriage						
Noncarrier						
Carrier	3.607	1.49–8.732	0.004*	2.805	1.641–4.794	<0.0001*
Age	0.865	0.811–0.923	<0.0001	1.023	0.998–1.049	0.07*
Gender						
Male	0.483	0.191–1.218	0.123	2.263	1.116–4.589	0.024*
Female						
Ethnicity						
Hindu	2.285	0.995–5.247	0.051*	0.714	0.424–1.203	0.206
Muslim						
Smoker	1.263	0.437–3.646	0.666	9.461	4.588–19.51	<0.0001*
Tobacco Chewer	0.187	0.05–0.695	0.012	3.562	2.103–6.032	<0.0001*
Tea Drinker						
≥4 cups/day	1.946	0.644–5.882	0.238	1.204	0.68–2.133	0.524
≤4 cups/day	1.045	0.293–3.719	0.946	0.406	0.192–0.862	0.019
Never						
-857T allele carriage						
Non-carrier						
Carrier	0.782	0.356–1.72	0.541	1.423	0.858–2.36	0.172
Age	0.864	0.811–0.922	<0.0001	1.019	0.994–1.044	0.142
Gender						
Male	0.568	0.233–1.383	0.212	2.378	1.19–4.752	0.014*
Female						
Ethnicity						
Hindu	2.068	0.924–4.628	0.077	0.688	0.413–1.144	0.15
Muslim						
Smoker	1.394	0.484–4.014	0.538	9.658	4.751–19.631	<0.0001*
Tobacco Chewer	0.225	0.063–0.805	0.022	3.675	2.192–6.161	<0.0001*
Tea Drinker						
≥4 cups/day	1.883	0.653–5.434	0.242	1.229	0.704–2.146	0.468
≤4 cups/day	1.067	0.314–3.634	0.917	0.395	0.189–0.823	0.013
Never						
-863A allele carriage						
Non-carrier						
Carrier	1.481	0.679–3.23	0.324	0.787	0.476–1.301	0.35
Age	0.866	0.813–0.922	<0.0001	1.019	0.994–1.044	0.134
Gender						
Male	0.584	0.24–1.422	0.236	2.374	1.189–4.741	0.014*
Female						
Ethnicity						
Hindu	1.93	0.865–4.307	0.109	0.703	0.423–1.17	0.175
Muslim						
Smoker	1.391	0.489–3.957	0.536	10.357	5.085–21.096	<0.0001*
Tobacco Chewer	0.221	0.062–0.793	0.021	3.728	2.224–6.249	<0.0001*
Tea Drinker						
≥4 cups/day	1.98	0.682–5.75	0.209	1.248	0.716–2.175	0.435
≤4 cups/day	1.049	0.308–3.58	0.938	0.394	0.189–0.82	0.013
Never						
-1031C allele carriage						
Non-carrier						
Carrier	1.729	0.764–3.908	0.189	2.537	1.483–4.342	0.001*
Age	0.865	0.812–0.921	<0.0001	1.021	0.996–1.047	0.102
Gender						
Male	0.588	0.241–1.434	0.243	2.362	1.17–4.769	0.016
Female						
Ethnicity						
Hindu	2.044	0.915–4.565	0.081	0.699	0.417–1.174	0.176
Muslim						
Smoker	1.359	0.477–3.87	0.566	9.28	4.522–19.044	<0.001*
Tobacco Chewer	0.221	0.061–0.794	0.021	3.6	2.133–6.084	<0.001*
Tea Drinker						
≥4 cups/day	1.897	0.651–5.531	0.241	1.224	0.693–2.161	0.486
≤4 cups/day	0.995	0.29–3.419	0.994	0.39	0.185–0.822	0.013
Never						

* Statistically significant (*P*<0.05).

## Discussion

Periodontitis is a multifactorial disease in which genetic and environmental factors affect the clinical outcomes. Among cytokines, TNF-α is considered as a pivotal mediator during the development of inflammatory response and the remodeling of periodontal tissue. In the present case–control study with a relatively large sample size, we investigated the possible role of the five different SNPs i.e. -238G/A, -308G/A, -857C/T, -863C/A, and -1031T/C located on TNF-α gene promoter region. Our case–control genetic association study was the first undertaken in an Indian population. Our study found that:

1. TNF-α -308G/A (rs1800629) genotype was significantly higher in patients with both AgP and CP compared with healthy controls;
2. TNF-α -857C/T (rs1799724) and -1031T/C (rs1799964) genotypes were significantly higher in patients with CP compared with healthy controls;
3. no association was found between TNF-α -238 G/A (rs361525) and -863 C/A (rs1800630) polymorphisms with susceptibility to AgP or CP.
4. In the AgP population, -238G/A (rs361252) and -308G/A genotypes were linked and had a high probability of coinheriting. In the CP population, -308G/A and -1031T/C (rs1799964) polymorphisms were linked and possibly coinherited in the next generation.
5. gender, smoking habit, and use of chewing tobacco increased the risk of CP, whereas in the AgP group there are no epidemiological factors that affect the risk of AgP.

As periodontitis is multifactorial in nature, involving interactions between the genes, the environment and lifestyle, periodontal risk gene assessments may be valuable in preventive, diagnostic, and therapeutic strategies against the incidence and progression of CP. There are different transition variants in the promoter region at the positions of -238, -308, -376, -857, -863 and -1031. It has been reported that the transition of guanine to adenine at -238, -308 position causes the high transcriptional activation which results enhanced production of TNF-α up to five-fold *in vitro* [25,30]. Other TNF-α SNPs at positions of -1031, -863, and -857 have been identified and the allelic variants of these SNPs have been suggested to be related to high TNF-α production [4,6]. However, the influence of different TNF-α genotypes on the phenotypic cytokine production is not fully known. The possible effect of TNF-α gene promoter polymorphism on the periodontal disease severity and clinical outcome remains to be elucidated.

Some studies have been conducted to evaluate the association between TNF-α promoter polymorphisms and periodontitis in different populations [23,26], but it is still a contradictory topic of debate. There are several contretemps regarding the TNF-α gene as a good candidate for genetic studies in relation to periodontitis. There is affirmation to suggest that TNF-α gene plays an important role in the pathogenesis of periodontitis as it is a potent immunological intermediator with proinflammatory properties [31]. TNF-α increases the rate of bone resorption and regulates cell proliferation from periodontal tissue origin [32,33]. It is determined that the interindividual differences have been observed in TNF-α production by peripheral blood mononuclear cells or oral leukocytes, isolated from individuals with and without periodontitis [34–36]. It is imaginable that individual differences in periodontitis susceptibility or individual differences in periodontal disease severity are related to genetically determined differences in TNF-α production and secretion by a variety of cells. The -238 polymorphism was shown to be nonfunctional in promoter/reporter gene studies [35,37]. On the other hand, it has been shown that carriers of the TNF-α -308 A allele appeared to have greater transcription activity and produced higher levels of TNF-α [38–40]. TNF-α levels are increased in the gingival cervicular fluid in periodontitis and this cytokine is found in higher levels in the inflamed periodontal tissues compared with healthy periodontal tissues [41].

In conclusion, our data show that the TNF-α -308G/A gene polymorphism is significantly associated with AgP, while -308G/A, -857C/T, and -1031T/C polymorphism increase the risk for CP in Indian population. Further studies including ethnically and culturally different populations from India confirm this finding.

## Abbreviations

AgP: aggressive periodontitis
CAL: clinical attachment loss
CP: chronic periodontitis
GI: gingival index
HC: healthy control
ICAM: intercellular adhesion molecule
MMP: matrix metalloproteinase
PD: probing depth
PI: plaque index
PMN: polymorphonuclear leukocyte
SNP: single nucleotide polymorphism
TNF-α: tumor necrosis factor-α
